# Hyperphosphatasia With Intellectual Disability (Mabry Syndrome) due to Compound Heterozygous *PIGO* Variants: First Molecularly Characterized Case in French Guiana: A Case Report and Review of the Literature

**DOI:** 10.1155/crig/2289202

**Published:** 2026-10-19

**Authors:** Mody Diop, Joddy Mafema Missindu, Narcisse Elenga

**Affiliations:** ^1^ Department of Medical Genetics University Hospital of French Guiana (Andrée Rosemon Hospital) Cayenne French Guiana; ^2^ Department of Pediatric Emergency Medicine University Hospital of French Guiana (Andrée Rosemon Hospital) Cayenne French Guiana; ^3^ Department of Pediatrics University Hospital of French Guiana (Andrée Rosemon Hospital) Cayenne French Guiana

**Keywords:** alkaline phosphatase, case report, developmental delay, GPI-anchor biosynthesis, *PIGO*, whole-exome sequencing

## Abstract

**Introduction:**

Hyperphosphatasia with intellectual disability syndrome (HPMRS), also known as Mabry syndrome, is a rare autosomal recessive disorder of glycosylphosphatidylinositol (GPI) anchor biosynthesis. Approximately 20 cases with pathogenic *PIGO* variants have been reported worldwide. This makes *PIGO*‐related HPMRS one of the rarest molecularly defined subtypes of the disease.

**Case Report:**

We report a male infant from French Guiana. He presented with neonatal tremors, global developmental delay with postural delay, and generalized tonic–clonic seizures with dystonic features. Symptoms began at 9 months and worsened by 13 months. Biochemical testing showed persistently elevated serum alkaline phosphatase (ALP 477 then 415 U/L; reference range 142–335 U/L). Trio whole‐exome sequencing identified two compound heterozygous *PIGO* variants: c.839T > *C* (p.Met280Thr), inherited from the father, and c.590C > *T* (p.Pro197Leu), inherited from the mother. The diagnostic laboratory classified both as probably pathogenic (Class 4). Physical examination at 17 months showed growth retardation (height and weight at −2 SD) and a reducible umbilical hernia. There was no notable facial dysmorphism or limb anomaly.

**Conclusion:**

We report the first molecularly characterized case of *PIGO*‐related HPMRS in French Guiana. The diagnosis combined clinical, biochemical, and molecular evaluation. This case shows that the *PIGO* phenotype extends beyond classic dysmorphic presentations.

## 1. Introduction

In 1970, Mabry et al. described a sibship with severe intellectual disability [1]. They also found a puzzling biochemical finding: markedly elevated serum alkaline phosphatase (ALP), with no identifiable bone or liver disease. This finding opened a window onto a broader group of genetic disorders. These disorders share a common molecular fault: defective synthesis or remodeling of the glycosylphosphatidylinositol (GPI) anchor. The GPI anchor is a lipid structure that tethers more than 150 proteins to the outer surface of the cell membrane [2]. This group is now known as hyperphosphatasia with intellectual disability syndrome (HPMRS). It has grown steadily as genomic sequencing has uncovered new causative genes.

The GPI pathway involves more than 30 genes working in sequence. ALP itself is one of the proteins anchored by it. This is why defects in this pathway produce a counterintuitive result: When ALP cannot reach the cell surface, it accumulates inside the cell and spills into the bloodstream in excess. Persistent isolated hyperphosphatasia is therefore a reliable and accessible biomarker for HPMRS, even before genetic testing is ordered [3]. Causative variants for this broader group of inherited GPI deficiencies (IGDs) have been identified in numerous genes. These include *PIGV*, *PIGN*, *PIGL*, *PGAP2*, *PGAP3*, *PIGG*, and phosphatidylinositol‐glycan biosynthesis class O (*PIGO*), among others. Hyperphosphatasia is not a universal feature of this group. It is characteristically absent in *PIGN*‐ and *PIGG*‐related disease. Inheritance is autosomal recessive for all subtypes [4].

*PIGO* encodes a protein involved in GPI anchor maturation. It transfers ethanolamine phosphate to the third mannose residue of the GPI glycan, a critical step that occurs in the endoplasmic reticulum [5]. Pathogenic *PIGO* variants cause HPMRS Type 2 (OMIM #614749). Krawitz et al. first described this condition in 2012. A recent systematic literature synthesis found approximately 20 reported cases since then [6]. The full phenotypic spectrum remains incompletely characterized [5]. Some patients also have brachytelephalangy, nail hypoplasia, or visceral anomalies. These features blur the clinical boundary with other CDG‐related syndromes, including Fryns syndrome [7, 8].

French Guiana presents a particular challenge for rare disease diagnosis. The territory has a highly diverse population of approximately 310,000 people, spread across an Amazonian territory twice the size of metropolitan Brittany. It has long lacked resident medical genetics expertise. Most families who eventually reach a specialist do so late, after a long diagnostic odyssey. We report here the first molecularly characterized case of *PIGO*‐related HPMRS in French Guiana. The diagnosis was reached through trio whole‐exome sequencing. We discuss its clinical, biological, and genetic characteristics.

## 2. Case Report

### 2.1. Clinical Presentation

The patient is a boy born at term (39 weeks and 5 days) to nonconsanguineous parents of Afro‐Caribbean Guianese origin. Delivery was induced by vaginal route for decreased fetal movements and cardiotocographic anomalies. Birth parameters were normal (weight 3910 g, height 50 cm, head circumference 35 cm; Apgar 10/10/10). This was the parents’ first child. No consanguinity was identified.

Parental concern began in the immediate neonatal period. While still in the maternity ward, the mother reported persistent cries of discomfort. The medical team initially attributed these to normal neonatal distress. After returning home, she observed repeated episodes of generalized clonus and trembling, sometimes with upward gaze deviation. These were not subtle findings. As she later told the CAMSP (Centre d’Action Médico‐Sociale Précoce) team, “he was not normal.” At Day 15 of life, the patient was admitted to the neonatal intensive care unit of the CHAR (Centre Hospitalier Andrée Rosemon, Cayenne) for abnormal movements. He remained there until Day 56. A broad metabolic workup was performed, including plasma and CSF amino acids, an acylcarnitine profile, and thyroid function. Results were essentially normal. Two EEGs showed no epileptiform activity. Brain MRI was unremarkable. G6PD deficiency was identified during this admission. Neonatal jaundice resolved without phototherapy.

The diagnostic odyssey that followed spanned 13 months and four institutions. After discharge, the patient received home hospitalization support and began physiotherapy for axial hypotonia and peripheral hypertonia. A remote neurology opinion was sought from Hôpital Trousseau in Paris. This team proposed pyridoxine dependency, malonic aciduria, hyperekplexia, and benign neonatal tremors as working diagnoses. All were ruled out: Trials of vitamin B6 and folinic acid produced no clinical improvement. Prof. Roubertie’s neuropediatric team in Montpellier was also consulted remotely. No specific diagnosis could be proposed. At 9 months, the patient had his first generalized tonic–clonic seizure (exact date not documented). This event prompted an urgent consultation with Dr. Kassimi, a neuropediatrician at the Centre Hospitalier de l’Ouest Guyanais (CHOG) in Saint‐Laurent du Maroni. Dr. Kassimi documented ongoing axial hypotonia, absent sitting, absent babbling, and persisting daily tremor episodes and recommended exome sequencing. At 11 months, the CAMSP Kayenn team in Cayenne began a multidisciplinary developmental follow‐up, including physiotherapy, occupational therapy, speech therapy, and psychology. Possible brachytelephalangy and nail hypoplasia were raised in the referral documentation. Clinical examination did not confirm these findings prominently. Despite this coordinated network of care, no diagnosis had been reached. His mother continued to massage him every morning and evening. This was the only gesture she found that reduced his discomfort.

At 13 months, the situation became more acute. Seizure frequency worsened. The patient was brought to the pediatric emergency department of the Centre Hospitalier Universitaire de Guyane (CHU de Guyane) in Cayenne, after a further generalized tonic–clonic seizure, now with dystonic posturing. The attending emergency physician, Dr. Joddy Mafema Missindu, immediately recognized the diagnostic complexity of the case. He referred the patient for urgent genetic evaluation. The patient’s mother was 13 weeks pregnant at the time of this referral. The urgency was therefore twofold: establish a diagnosis for the patient and assess the recurrence risk in time for the ongoing pregnancy. Treatment was started with levetiracetam (Keppra) for seizure control and trihexyphenidyl (Artane) for the dystonic component. Seizure frequency partially decreased but was not fully controlled. A follow‐up EEG performed after this episode was normal.

At last follow‐up, the patient was approximately 3 years old. Seizures were controlled on the current antiepileptic regimen. He attends the CAMSP for physiotherapy, psychomotor therapy, and occupational therapy. He has achieved independent sitting. He has not yet crawled on all fours and does not yet produce words.

### 2.2. Physical Examination at 17 Months

We examined the patient at 17 months, during an outreach genetics mission to Cayenne (October 2024). His growth was uniformly retarded: height 75 cm (−2 SD), weight 8.9 kg (−2 SD), and head circumference 47 cm (−1 SD).

We found no facial dysmorphism and no anomaly of the hands or feet. Cardiac and pulmonary auscultation were normal. The abdomen was soft, with no hepatosplenomegaly. A reducible umbilical hernia with a wide neck was the only structural anomaly identified. Both testes were palpable in the scrotum. There was no sacral dimple. The patient’s social engagement struck us most during the examination: He smiled, babbled, and tracked faces. This suggested that his affective and social capacities were relatively preserved despite his significant motor delay. No standardized developmental assessment tool was used; developmental status was based on clinical observation alone. Some episodes of posterior axial hypertonia were observed after feeding.

### 2.3. Biochemical Investigations

Faced with unexplained global developmental delay and a movement disorder in a child with nonspecific neurological investigations, we measured serum ALP. The result came back on two separate occasions, at two different laboratories:

• First measurement (Biomnis laboratory): ALP = 477 U/L (reference range 142–335 U/L)
• Second measurement (Laboratoire de Biologie Polyvalente, CHC Andrée Rosemon, Cayenne): ALP = 415 U/L (reference value < 281 U/L for age)

Published age‐specific pediatric reference intervals, derived from large‐scale percentile analyses, show that ALP activity is physiologically higher in infancy than later in childhood, before declining toward the ranges used by both laboratories here [9]. Against this background, both values were clearly and consistently above the upper limit of normal for a child of this age. This makes a transient or purely laboratory‐related elevation unlikely. The lower reference bound reported by the second laboratory (< 281 U/L) could not be independently verified against published percentile data and should be confirmed with the laboratory. Contemporaneous GGT, bilirubin, phosphate, PTH, and vitamin *D* were not available. Cholestatic, hyperparathyroid, or metabolic bone causes could therefore not be formally excluded biochemically. None were suggested clinically.

### 2.4. Molecular Genetic Investigations

We sent blood samples from the patient and both parents to the Eurofins Biomnis laboratory in Lyon (collected July 16, 2024) for trio whole‐exome sequencing. The analysis used the Human Exome 2.0 Plus Comprehensive Exome capture kit with Illumina NOVASEQ 6000 sequencing (150 bp paired‐end reads; > PIGG 98% bases covered at ≥ 10X; 45 million read pairs). Variant interpretation followed ACMG criteria. Results were returned 3 months later, in October 2024. By then, the patient was 17 months old, his mother was in the third trimester of pregnancy, and the family had been waiting for a diagnosis for over a year.

The primary analysis identified a positive result. The proband carries two compound heterozygous variants in *PIGO* (NM_032634.4). The diagnostic laboratory classified both as probably pathogenic (Class 4; Table 1). Segregation of the two variants in the family is shown in the pedigree (Figure 1).

**TABLE 1:** Compound heterozygous *PIGO* variants identified by trio whole‐exome sequencing.

Gene	Variant (cDNA)	Protein	Zygosity	Origin	Laboratory classification
*PIGO*	c.839T > *C*	p.Met280Thr	Heterozygous	Paternal	Class 4 (Prob. pathogenic)
*PIGO*	c.590C > *T*	p.Pro197Leu	Heterozygous	Maternal	Class 4 (Prob. pathogenic)

*Note:* Both variants were classified as probably pathogenic (Class 4) by the diagnostic laboratory’s five‐tier system. Our independent ACMG/AMP review, detailed below, confirms Likely Pathogenic status for c.839T > *C*. The evidence for c.590C > *T* falls short of this threshold.

**FIGURE 1:**
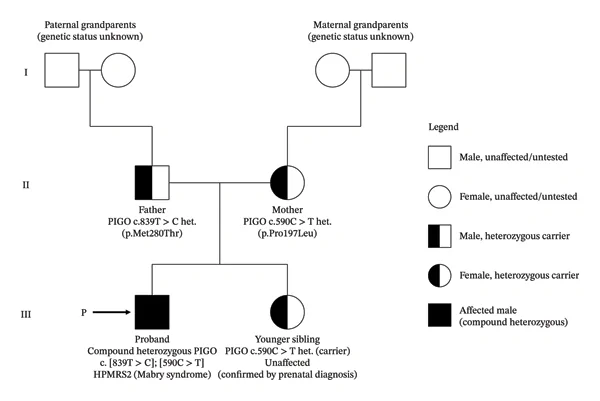
Pedigree of the family (identifying details removed). Generation I: grandparents (genetic status unknown). Generation II: father (heterozygous carrier of *PIGO* c.839T > *C*, p.Met280Thr) and mother (heterozygous carrier of *PIGO* c.590C > *T*, p.Pro197Leu). Generation III: proband (affected, compound heterozygous *PIGO*) and a younger sibling, confirmed heterozygous carrier by prenatal diagnosis and clinically unaffected. Square = male, circle = female. Filled symbol = affected individual. Half‐filled symbol = confirmed heterozygous carrier (unaffected). Open symbol = unaffected or untested individual. Symbols follow standard pedigree conventions and remain distinguishable in grayscale (sex = shape, carrier or affected status = fill, independent of color).

The c.839T > *C* (p.Met280Thr) variant is in the catalytic domain of the *PIGO* protein. *PIGO* is a GPI‐ethanolamine phosphate transferase that adds the terminal phosphoethanolamine residue to the third mannose of the GPI anchor [5]. This variant has been observed 13 times in the heterozygous state, but never in the homozygous state, in population databases (gnomAD v4.1, allele frequency 8.1 × 10^−6^). It is currently reported as a variant of uncertain significance in ClinVar (single submission), and it is absent from HGMD. In silico pathogenicity tools support a deleterious effect (CADD 26.2; REVEL 0.92).

We applied ACMG/AMP criteria as follows. PM2: absence in the homozygous state, low population frequency. PM3: confirmed trans configuration with a second *PIGO* variant in a recessive disease, paternal origin. PP3: computational evidence supports a deleterious effect. PP4: the phenotype is specific for a disease with a single genetic etiology. This combination meets the threshold generally used for Likely Pathogenic under standard ACMG/AMP combining rules. The same variant was independently classified as Likely Pathogenic (PM2_Moderate, PP3_Moderate, PP4_Moderate) in an unrelated patient reported by Starosta et al. [7]. This provides external corroboration.

The c.590C > *T* (p.Pro197Leu) variant is also in the catalytic domain. It has a comparable population frequency (gnomAD v4.1, 14 heterozygotes, 0 homozygotes, allele frequency 8.7 × 10^−6^). HGMD Professional reports it as pathogenic, in association with epilepsy. ClinVar currently classifies it as a variant of uncertain significance (multiple submissions). We could not independently resolve this discrepancy between sources. In silico predictions do not support a deleterious effect (CADD 26.6; REVEL 0.37, below the threshold generally used to support pathogenicity).

We applied the same ACMG/AMP criteria. PM2 and PM3 are met: low population frequency and confirmed trans configuration with maternal origin. PP4 applies at the family level but is not variant‐specific. Two Moderate criteria with one Supporting criterion fall short of the threshold generally required for Likely Pathogenic under standard ACMG/AMP combining rules. We therefore classify this variant as probably pathogenic on the diagnostic laboratory’s five‐tier system. We do not independently reclassify it as ACMG/AMP Likely Pathogenic on the evidence available to us.

ACMG/AMP Likely Pathogenic status is therefore more firmly established for c.839T > *C* than for c.590C > *T*. We describe the molecular findings as consistent with *PIGO*‐related HPMRS, rather than as a definitive diagnosis of HPMRS2. The patient carries a heterozygous, ACMG/AMP‐confirmed likely pathogenic *PIGO* variant (c.839T > *C*) in trans with a second heterozygous *PIGO* variant of more uncertain individual significance (c.590C > *T*). Combined with persistent hyperphosphatasia, developmental delay, and early‐onset epilepsy, this molecular and clinical picture is consistent with *PIGO*‐related HPMRS (Mabry syndrome, OMIM #614749). No functional studies have been performed for either variant in this family.

### 2.5. Prenatal Diagnosis

When the *PIGO* diagnosis was confirmed, the patient’s parents were informed that no curative treatment exists for this condition. Both parents had given up their studies to care for the patient. By the time of diagnosis, approximately 14 months had elapsed since the initial presentation. The molecular diagnosis had two main clinical benefits: It explained the phenotype accurately, and it gave actionable information for the ongoing pregnancy. Both parents carry one pathogenic *PIGO* variant each, so the recurrence risk was 25% for any future pregnancy. Prenatal molecular diagnosis was offered and immediately accepted. Amniocentesis was performed at 20 weeks of gestation, with targeted testing for the two familial *PIGO* variants. Maternal‐cell contamination was excluded according to the testing laboratory’s standard protocol. The result brought significant relief: The fetus carried only the maternal c.590C > *T* (p.Pro197Leu) variant, in the heterozygous state, and would not be affected. The mother completed her pregnancy without further complication and delivered safely.

### 2.6. Comparison With Previously Reported *PIGO* Cases

To put the patient’s presentation in context, we compared his clinical, biochemical, and molecular findings with those of previously published individuals with molecularly confirmed *PIGO*‐related disease. We identified these cases through a targeted literature search (Table 2).

**TABLE 2:** Comparison of the present patient with previously reported PIGO cases.

Reference	PIGO variant (s)	Age	ALP	Neurodevelopmental phenotype	Dysmorphism/malformations	Outcome
Krawitz et al. 2012, Am J Hum Genet 91 (1):146–151	Family A (variants not detailed in available extracts); one further isolated case	Examined at 12–15 years (Family A)	Elevated (diagnostic criterion of the index series)	Intellectual disability	Facial dysmorphism; nail hypoplasia (Digits 2 and 4, absent nail of Digit 5); broad hallux; hypoplastic toenails	Not reported
Nakamura et al. 2014, Epilepsia 55: e13‐e17	Not detailed in available extracts	Not specified	Only mildly elevated	Intractable epilepsy, severe developmental delay	Not detailed	Not reported
Zehavi et al. 2017, Metab Brain Dis 32: 2131–2137	c.765G > *A*, p.(Met255Ile), homozygous (consanguineous family)	Not specified (2 sisters)	Normal, despite severe phenotype	Severe infantile epileptic encephalopathy, intractable seizures, axial hypotonia	Low‐set ears, hypertelorism, upslanting palpebral fissures, broad nasal bridge, blue sclerae; corpus callosum hypoplasia	Not reported
Starosta et al. 2023 (PIGO‐CDG), JIMD Rep 64: 424–433	Novel variants (not detailed in available extracts)	22 months (female)	Elevated	Global developmental delay	Classical Mabry phenotype plus functional hyperinsulinism/hypoglycemia, severe secretory diarrhea, osteopenia with pathological fracture	Extended, severe phenotype
Tanigawa et al. 2017, Hum Mutat 38 (7): 805–815^†^	5 patients (3 sibships): c.1109A > *G*(p.Asn370Ser)/*c*.2497_2498del and c.1109A > *G*/*c*.2496del; c.1031T > *A*(p.Met344Leu)/*c*.1288C > *T*(p.Gln430∗) (sibship)	Not specified (pediatric cohort)	Markedly elevated (2594–5229 U/L in 3/5 patients)	Global developmental delay in all; sensorineural deafness in 3/5; epilepsy in 2/5 (onset 2 years)	Facial dysmorphism (upslanting palpebral fissures, prominent nasal bridge, cleft lip/palate in 3/5); ichthyosis (3/5); nail hypoplasia (3/5); congenital megacolon (2/5)	2 early deaths reported (septicemia at 17 months; pneumonia at 18 months)
Dellepiane et al. 2024, Biomedicines 12 (12): 2779	c.839T > *C* (p.Met280Thr, paternal. SHARED VARIANT with the present patient); c.2285C > *T* (p.Pro762Leu, maternal, VUS)	Onset at Day 1 (abdominal distension); seizures from day 7	Markedly elevated (1689 U/L) with hyperphosphatasemia	Encephalopathy, drug‐resistant epilepsy, generalized hypertonicity; evolving MRI pattern (“mild” then “severe” within 1 month)	Brachydactyly, nail hypoplasia, facial dysmorphism (elongated palpebral fissures, preauricular pits); congenital aganglionic megacolon	Died at 6 months (comfort care after ethics committee review)
Wang et al. 2025, Front Pediatr 13: 1667477. Patient 1	c.2612A > *C* (p.His871Pro)/c.2361dup (p.Thr788Hisfs∗5)	5 years 2 months (delayed language)	600–900 U/L (fluctuating)	Isolated delayed language development in the foreground; hyperactivity; no seizures reported	Ear malformation, palmoplantar hyperkeratosis; hypertonia; MRI: widened extra‐axial spaces	Ongoing follow‐up, partial improvement with rehabilitation
Wang et al. 2025, Front Pediatr 13: 1667477. Patient 2	c.2510T > *A* (p.Val837Asp)/c.693C > *G* (p.Phe231Leu)	4 months 28 days (delayed motor/cognitive development)	523 U/L (mildly elevated)	Global motor and cognitive delay, involuntary movements, normal EEG	Triangular face, high nasal bridge; MRI: bilateral ventriculomegaly, delayed myelination	Ongoing follow‐up, unfavorable course despite rehabilitation
This report	c.839T > *C* (p.Met280Thr, paternal); c.590C > *T* (p.Pro197Leu, maternal), compound heterozygous	First seizure at 9 months; molecular diagnosis at 17 months	Moderately elevated (415–477 IU/L)	Global developmental delay, epilepsy (two seizures), dystonia	No marked facial dysmorphism; isolated umbilical hernia; no confirmed brachytelephalangy or nail hypoplasia	Follow‐up ongoing; prenatal diagnosis performed for the sibship (fetus unaffected)

*Note:* This comparison is based on a targeted literature search. It is not intended as an exhaustive systematic review. Several published cases report incomplete phenotypic detail in their abstracts.

^†^Direct full‐text access to Tanigawa et al. 2017 was not obtained (publisher access restriction). The data shown were cross‐verified against the genotype–phenotype table published in Wang et al. 2025, which tabulated these patients from the Tanigawa et al. full text.

### 2.7. Timeline of Care

Table 3 summarizes the diagnostic and clinical course described above, from birth to last follow‐up.

**TABLE 3:** Timeline of the diagnostic and clinical course, from birth to last follow‐up.

Timepoint	Event
Birth (term, 39 weeks 5 days)	Birth parameters normal (weight 3910 g, height 50 cm, head circumference 35 cm); Apgar 10/10/10.
Immediate neonatal period	Mother reports persistent cries of discomfort while still in the maternity ward.
After discharge home	Repeated episodes of generalized clonus and trembling observed, sometimes with upward gaze deviation.
Day 15 of life	Admitted to the neonatal intensive care unit (CHAR, Cayenne) for abnormal movements; broad metabolic workup, two EEGs, and brain MRI unremarkable; G6PD deficiency identified; neonatal jaundice resolved without phototherapy.
Day 56 of life	Discharged home; home hospitalization support and physiotherapy begun for axial hypotonia and peripheral hypertonia.
Remote specialist opinions (Paris, Montpellier)	Hôpital Trousseau team proposes pyridoxine dependency, malonic aciduria, hyperekplexia, and benign neonatal tremors; all ruled out (no improvement with vitamin B6 or folinic acid trials). Prof. Roubertie’s neuropediatric team (Montpellier) consulted; no diagnosis proposed.
9 months	First generalized tonic–clonic seizure; urgent consultation with Dr. Kassimi (CHOG, Saint‐Laurent du Maroni), who recommends exome sequencing.
11 months	Multidisciplinary developmental follow‐up begins at CAMSP Kayenn (Cayenne).
13 months	Seizure frequency worsens, now with dystonic posturing; urgent referral for genetic evaluation from the pediatric emergency department (CHU de Guyane); levetiracetam and trihexyphenidyl started; mother 13 weeks pregnant.
July 16, 2024	Trio blood samples sent to Eurofins Biomnis (Lyon) for whole‐exome sequencing.
17 months (results returned, October 2024)	Compound heterozygous *PIGO* variants identified by trio whole‐exome sequencing; outreach genetics physical examination performed in Cayenne.
20 weeks’ gestation (subsequent pregnancy)	Prenatal amniocentesis for the two familial *PIGO* variants; fetus found to carry only the maternal c.590C > *T* variant, heterozygous and unaffected.
Approximately 3 years (last follow‐up)	Seizures controlled on current antiepileptic regimen; independent sitting achieved; ongoing physiotherapy, psychomotor therapy, and occupational therapy at CAMSP.

## 3. Discussion

After confirming the patient’s diagnosis, we searched the literature. A subsequent systematic literature synthesis identified approximately 20 published patients carrying *PIGO* pathogenic variants worldwide. We could not identify any prior molecularly confirmed case from the Caribbean or South American region. This rarity shaped how we approached this report. We treat it not as a routine case description but as a contribution to a body of evidence thin enough that every patient genuinely matters.

### 3.1. Comparison With Previously Reported Cases and Diagnostic Evolution Over Time

Table 2 situates the present patient within the published *PIGO* literature. Global developmental delay is the only feature shared by every case in the table, including ours. Epilepsy is frequent but not constant. Facial dysmorphism, brachytelephalangy, and nail hypoplasia are inconsistent. They were prominent in the original family described by Krawitz et al. [5]. They are absent or unconfirmed in our patient and variably reported elsewhere. This variability argues against treating dysmorphism as a requirement for molecular testing, a point the referring clinicians in this case also raised.

Serum ALP was elevated in most reported cases, including ours, but not all. Zehavi et al. [10] described two sisters with a homozygous *PIGO* missense variant, severe infantile epileptic encephalopathy, corpus callosum hypoplasia, and persistently normal ALP throughout their course. This case qualifies a claim made elsewhere in this report: that ALP elevation reliably flags *PIGO*‐related disease early. ALP is a useful, accessible adjunct in most cases. However, a normal value does not exclude the diagnosis. The decision to test a child with a compatible phenotype should not depend on ALP alone.

The same paternal variant identified in our patient, c.839T > *C* (p.Met280Thr), was also reported by Dellepiane et al. [11]. Their patient was a term newborn with a different maternal allele (p.Pro762Leu, a variant of uncertain significance). That infant presented on Day 1 of life with congenital aganglionic megacolon, brachydactyly, facial dysmorphism, and a markedly elevated ALP (1689 U/L). His neuroradiological course progressed rapidly, and he died at 6 months. Our patient inherited the same p.Met280Thr allele, in trans with p.Pro197Leu. His course has been considerably milder to date: two seizures, moderate ALP elevation, and no dysmorphism or gastrointestinal malformation. This contrast supports a broader observation: The phenotype in *PIGO*‐related disease is shaped by the specific combination of alleles inherited in trans, not by either variant alone. It cautions against assuming a fixed severity for a given variant across families.

The published cases also span more than a decade. The gene was first linked to hyperphosphatasia with mental retardation syndrome in 2012, in a European academic center with direct access to exome sequencing [5]. Reports since then have broadened the recognized phenotype considerably. This ranges from the classical dysmorphic presentation to milder, purely neurodevelopmental forms like this one. At the other end, it includes forms with normal biochemistry [10] or multisystem involvement, including endocrine and gastrointestinal disease. Wang et al. [6] recently published a systematic literature synthesis, based on a documented PubMed search (“HPIDS2” OR “PIGO”, January 2001–December 2023). They tabulated genotype–phenotype data for 20 previously published patients. This work situates the phenotypic range summarized here within a more complete dataset. Despite more than a decade of accumulated experience, our patient’s diagnostic pathway in French Guiana in 2023–2024 still required the same basic steps as the earliest reports: clinical suspicion, biochemical testing, and exome sequencing performed thousands of kilometers away. The added delay came from distance to specialized genetic services. This structural delay, more than 10 years after the gene was identified, seemed worth documenting on its own.

Overall, this patient’s presentation sits toward the milder end of the reported spectrum. It combines moderate ALP elevation, epilepsy, dystonia, and global developmental delay, without confirmed dysmorphism or malformation. It is closer to isolated neurodevelopmental cases than to the multisystem phenotypes described in some series. We cannot tell from a single patient whether this reflects a genuinely milder effect of these variants, an incomplete assessment of subtle dysmorphic features at 17 months, or both. Longer follow‐up and more cases, ideally pooled through international registries, would help answer this question.

### 3.2. Hyperphosphatasia as a Diagnostic Key

Serum ALP was measured as part of the diagnostic workup initiated at the time of urgent genetic referral, in parallel with trio whole‐exome sequencing. The two ALP measurements were drawn several weeks after the exome sample had already been sent to Lyon. Even so, results were available well before the exome results. They provided early, corroborating biochemical evidence while the family awaited a molecular diagnosis. The mechanism is straightforward. ALP is normally exported to the cell surface through a GPI anchor. When GPI assembly fails, ALP accumulates inside the cell and leaks into the circulation as a soluble, hyperactive form [3]. This elevation is sensitive and can appear before the full clinical picture develops.

In a territory like French Guiana, whole‐exome sequencing requires referral to metropolitan France. Turnaround takes 4–6 months, with coordination across time zones. In this setting, the ability to flag a case early with a standard biochemistry panel remains valuable, even when, as in this case, it does not precede the decision to pursue genomic testing. The patient’s two independent ALP measurements (477 and 415 U/L, against a ceiling of 335 U/L) were consistently elevated. They reinforced the clinical suspicion of a GPI‐anchor disorder while exome results were pending. In settings with limited genetic medicine access, ALP measurement is a useful, low‐cost adjunct to the neurodevelopmental workup. It is best used in parallel with genomic testing, not as a prerequisite for it.

### 3.3. Genotype–Phenotype Considerations

The two *PIGO* variants add different kinds of evidence. The combination of epilepsy with only mildly elevated ALP has previously been described in *PIGO* deficiency [12]. This pattern broadly matches the patient’s trajectory. However, the variant reported by Nakamura et al. differs from either allele identified here. This should be read as a phenotype‐level parallel, not as evidence of an allele‐specific correlation for p.Pro197Leu specifically. The p.Met280Thr variant (c.839T > *C*), inherited from the father, has been less frequently reported. It carries a higher REVEL score (0.92 vs. 0.37), pointing toward a more disruptive effect on protein function.

Together, these two variants produced a phenotype dominated by developmental delay and epilepsy, the core features of Mabry syndrome. The dysmorphic component remained comparatively subtle. When we examined the patient at 17 months, we found no facial dysmorphism and no convincing limb anomaly. This was despite brachytelephalangy and nail hypoplasia having been raised as clinical questions during the laboratory referral process. Whether these features will appear later, or are simply absent in this patient, is not yet clear.

The absence of major dysmorphism in our patient sits at the milder end of a wide phenotypic spectrum. The previously described *PIGO* phenotypic spectrum ranges from classical Mabry syndrome (intellectual disability, epilepsy, brachytelephalangy, and facial dysmorphism) to multisystemic presentations approaching Fryns syndrome (with Hirschsprung disease, vesicoureteral anomalies, and anorectal malformations) [7, 8, 13]. Our patient’s presentation falls somewhere in between, closer to the mild end: no major organ malformation and only subtle external dysmorphism. The only visceral finding was an umbilical hernia, a fairly nonspecific anomaly. This variability is a reason not to exclude *PIGO*‐related HPMRS on the basis of a normal‐looking child.

### 3.4. A First in French Guiana: Implications for Health Equity

This diagnosis did not happen because French Guiana has a rare disease infrastructure in place. It happened without one. It depended on several things being available at the same time: local biochemical testing in Cayenne, exome sequencing capacity in Lyon, a multidisciplinary variant review in Paris, and an outreach genetics consultation on site. If any one of these had been missing, the patient would probably still be undiagnosed. This dependence on external, resource‐intensive coordination directly contributed to the patient’s 17‐month interval between first symptom and molecular diagnosis. It argues for durable local investment in genetic testing capacity to shorten that interval for future patients.

Similar access gaps in genetic medicine likely affect neighboring territories with comparable population structures. This includes Martinique, Guadeloupe, Suriname, and the wider Amazonian region. HPMRS may be equally underdiagnosed there and equally detectable by routine ALP testing.

## 4. Conclusion

This patient is, as far as we know, the first person with molecularly characterized *PIGO* deficiency diagnosed in French Guiana. He presented with neonatal tremors, followed by a long period of unexplained global developmental delay, before reaching a genetic diagnosis at 17 months. This course shows what systematic biochemical screening can achieve. It also shows how much delay results when specialist expertise is not available locally.

Two points from this case are worth keeping in mind. First, serum ALP is a cheap, widely available test that remains underused in the workup of unexplained developmental delay. It can flag a GPI‐anchor deficiency months before exome results return. Second, this patient’s phenotype, severe epilepsy and global developmental delay with minimal dysmorphism, shows that a normal‐looking child should not be excluded from molecular testing on that basis alone. The *PIGO*‐related phenotype is broader than the classical description suggests.

Reaching this diagnosis required a pediatric hospitalization in Cayenne, two ALP assays, an exome sent to Lyon, a multidisciplinary staff meeting, and a genetics outreach mission from Paris. This combination of resources led to a diagnosis in this instance. However, diagnostic pathways for rare diseases should not depend on this specific set of circumstances being available. Developing sustainable local genetic medicine capacity in French Guiana, and in comparable overseas territories, is a clinical and public health priority for the families currently awaiting a diagnosis.

## Author Contributions

Mody Diop: clinical genetics evaluation, molecular data interpretation, genetic counseling, and manuscript drafting and revision. Joddy Mafema Missindu: initial emergency clinical assessment, patient referral, and review of the manuscript. Narcisse Elenga: pediatric clinical follow‐up, multidisciplinary case management, and review and approval of the final manuscript.

## Funding

This case report received no specific funding from any funding agency in the public, commercial, or not‐for‐profit sectors.

## Disclosure

All authors read and approved the final manuscript.

## Ethics Statement

This case report describes a single patient and does not constitute human subjects research under institutional policy; therefore, ethics committee/institutional review board approval was not required. Written informed consent for publication of this case report, including the clinical, genetic, and pedigree information and the accompanying figure, was obtained from both parents prior to submission.

## Conflicts of Interest

The authors declare no conflicts of interest.

## Patient Perspective

The patient’s parents shared that the months preceding diagnosis were marked by sustained uncertainty: both had left their studies to care for him, and his mother described her son’s early distress as behavior that “was not normal,” a concern the family raised repeatedly before a diagnosis was reached. They found that a daily massage routine, performed each morning and evening, was the only measure that reliably eased his discomfort. The parents consented to the publication of this report in the hope that it might help other families and clinicians facing a similar diagnostic journey.

## Supporting Information

Additional supporting information can be found online in the Supporting Information section.

## Supporting information


**Supporting Information** CARE Checklist—completed.

## Data Availability

The *PIGO* variants reported in this study have been submitted to ClinVar. Additional clinical and molecular data supporting the findings of this case are available from the corresponding author upon reasonable request, subject to the constraints of patient confidentiality. This report follows the CARE case report guidelines. A completed CARE checklist is available from the corresponding author.
