# Supplementary material for: Hyperphosphatasia With Intellectual Disability (Mabry Syndrome) due to Compound Heterozygous PIGO Variants: First Molecularly Characterized Case in French Guiana: A Case Report and Review of the Literature
Source: Case Rep Genet. 2026 Oct 19;2026:2289202. doi: 10.1155/crig/2289202 (PMC13680857; doi:10.1155/crig/2289202)
Supplement: Supplementary file 1 [file CRIG-2026-2289202-s001.docx]

**CARE Checklist – Completed**

**Manuscript 2289202:** Hyperphosphatasia With Intellectual Disability (Mabry Syndrome) due to Compound Heterozygous *PIGO* Variants: First Molecularly Characterized Case in French Guiana: A Case Report and Review of the Literature

Mody Diop, Joddy Mafema Missindu, Narcisse Elenga

Based on the 2013 CARE Guidelines 13-item checklist (care-statement.org).

| **No.** | **Topic** | **Item** | **Reported? / Location in manuscript** |
| --- | --- | --- | --- |
| 1 | Title | The diagnosis or intervention of primary focus followed by the words “case report”. | Yes — Title: “…First Molecularly Characterized Case in French Guiana: A Case Report and Review of the Literature”. |
| 2 | Key Words | 2 to 5 key words that identify diagnoses or interventions in this case report (including “case report”). | Yes — Keywords: PIGO; GPI-anchor biosynthesis; alkaline phosphatase; developmental delay; whole-exome sequencing; case report. |
| 3a | Abstract | Introduction – What is unique about this case and what does it add to the scientific literature? | Yes — Abstract, “Introduction” subsection. |
| 3b | Abstract | The patient’s main concerns and important clinical findings. | Yes — Abstract, “Case Report” subsection. |
| 3c | Abstract | The primary diagnoses, interventions, and outcomes. | Yes — Abstract, “Case Report” subsection. |
| 3d | Abstract | Conclusion – What are one or more “take-away” lessons from this case report? | Yes — Abstract, “Conclusion” subsection. |
| 4 | Introduction | Briefly summarizes why this case is unique and may include medical literature references. | Yes — Section 1 (Introduction). |
| 5 | Patient Information | De-identified patient specific information. | Yes — Section 2.1 (Clinical Presentation) and Figure 1 (pedigree, identifying details removed). |
| 5 | Patient Information | Primary concerns and symptoms of the patient. | Yes — Section 2.1, paragraph 2 (parental concern, neonatal tremors, clonus). |
| 5 | Patient Information | Medical, family, and psychosocial history including relevant genetic information. | Yes — Section 2.1 (non-consanguineous parents, first child) and Figure 1 (pedigree); Section 2.5 (parental carrier status, recurrence risk). |
| 5 | Patient Information | Relevant past interventions and their outcomes. | Yes — Section 2.1, paragraph 3 (trials of vitamin B6 and folinic acid; remote neurology opinions). |
| 6 | Clinical Findings | Describe significant physical examination (PE) and important clinical findings. | Yes — Section 2.2 (Physical Examination at 17 Months). |
| 7 | Timeline | Historical and current information from this episode of care organized as a timeline (figure or table). | Yes — Section 2.7 (Timeline of Care) and Table 3, from birth to last follow-up. |
| 8 | Diagnostic Assessment | Diagnostic methods (PE, laboratory testing, imaging, surveys); diagnostic challenges; diagnosis (including other diagnoses considered); prognostic characteristics when applicable. | Yes — Section 2.1 (differential diagnoses considered during the diagnostic odyssey), Section 2.3 (Biochemical Investigations) and Section 2.4 (Molecular Genetic Investigations, including ACMG/AMP classification). |
| 9 | Therapeutic Intervention | Types, administration, and changes in therapeutic intervention. | Yes — Section 2.1, paragraph 4 (levetiracetam and trihexyphenidyl; seizure control). |
| 10 | Follow-up and Outcomes | Clinician- and patient-assessed outcomes; follow-up test results; intervention adherence/tolerability; adverse and unanticipated events. | Partial — Follow-up status and outcome in Section 2.1 (last paragraph, approximately age 3 years) and Section 2.5 (prenatal diagnosis outcome). Adverse or unanticipated events are not separately reported. |
| 11 | Discussion | Strengths and limitations; relevant medical literature; rationale for conclusions; primary take-away lessons. | Yes — Section 3 (Discussion), Subsections 3.1–3.4. |
| 12 | Patient Perspective | The patient (or parents, given the patient’s age) should share their perspective on the care received. | Yes — Dedicated “Patient Perspective” section. |
| 13 | Informed Consent | The patient (here, the parents) should give informed consent for publication. | Yes — Ethics Statement and Acknowledgments (written informed consent obtained from both parents). |
